# Impact of Medical Student Involvement on Emergency Department Outcomes: A Tertiary Center Analysis

**DOI:** 10.5811/westjem.42229

**Published:** 2025-07-08

**Authors:** Ryan Ballard, Asfia Qureshi, Chengu Niu, Keith Grams, Mathew Devine, Nagesh Jadhav, Richard Alweis

**Affiliations:** * Lake Erie College of Osteopathic Medicine, Erie, Pennsylvania; † Rochester Regional Health, Department of Medicine, Rochester, New York; ‡ Rochester Regional Health, Department of Emergency Medicine, Rochester, New York; § Rochester Regional Health, Department of Medical Education, Rochester, New York

## Abstract

**Introduction:**

Increasing patient use of emergency departments (ED) and overcapacity threaten both efficiency of the care provided and the teaching mission. We investigated the influence of medical student (MS) involvement on ED throughput, resource use, and clinical outcomes, and we addressed gaps in existing literature that primarily focus on resident physicians and singular throughput metrics.

**Methods:**

We conducted a retrospective observational analysis of 123,503 encounters with patients >21 years of age at an urban, tertiary-care hospital, comparing cases with and without MS participation. We excluded patients seen by advanced practice practitioners. We compared continuous variables using *t*-tests with bootstrap, and categorical variables by chi-square tests. Continuous variables were reported with mean and standard deviation.

**Results:**

We analyzed patient encounters both with and without MS coverage across various complexity levels from January 1, 2022–December 31, 2023. Of the 123,503 patient encounters, 9,635 (7.8%) involved MS participation, and 113,868 (92.2%) did not. Across all encounters, door-to-physician time showed no significant difference between encounters with (28.1 minutes ± 38.6) and without medical students (28.4 minutes ± 38.0; *P* = .435), while door-to-triage and arrival-to-disposition time (292.6 minutes ± 193.7 vs 270.4 minutes ± 532.8; *P* < .001) and doctor-to-disposition time (266.8 minutes ± 186.1 vs. 242.9 minutes ± 376.4; P < .001) were significantly longer. In high-complexity encounters, patients seen with medical students experienced shorter door-to-physician (26.6 vs 28.2 minutes, *P* < .001), door-to-triage (13.6 vs 14.5 minutes, P = .03), arrival-to-disposition (301.1 vs 307.7 minutes, *P* = .02), and doctor-to-disposition times (275.2 vs 281.3 minutes, *P* =.02).

**Conclusion:**

We found that medical student involvement is associated with longer patient stays in low- to medium-complexity cases but improved efficiency in the management of high-complexity cases. Increased rates of some diagnostic imaging and higher admission rates occurred with medical students. Our single-center design highlights the need for multicenter validation of these findings to inform future resource allocation and educational strategies in the ED.

## INTRODUCTION

With healthcare access shortages, the role of the emergency department (ED) has evolved from its original mission of stabilizing and treating critically ill patients to encompass a broader function as a safety net for primary medical care.^1^,^2^ This shift in medical infrastructure has significantly impacted the efficiency of care provided within the ED, contributing somewhat to crowding^3^,^4^ with the acknowledgement that hospital capacity constraints remain the primary driver of ED crowding.^5^,^6^ In this milieu of meeting multiple community and hospital needs, rotations within the ED remain an integral facet of a medical student’s (MS) educational development.^7^ Consequently, MS involvement in patient care requires that emergency physicians dedicate additional time on shift toward student presentations, supervision, and teaching. While some studies have highlighted improved patient outcomes with MS participation, concerns persist regarding potential inefficiencies in clinical care environments that are inherently reliant on timely intervention.^8^–^11^ The challenge posed to hospitals, and to EDs in particular, is how to properly balance an academic mission with high-quality care, timely throughput, and satisfactory patient metrics.

Previous investigations have attempted to quantify the impact of a learner on patient care, although these studies have primarily involved smaller cohorts and often used a singular throughput metric (eg, ED length of stay [LOS]).^12^–^15^ A recent systematic review rated these studies as low-quality evidence with high risk of bias, highlighting the difficulty of isolating MS effects on the provision of care in a multicare model department.^16^ While LOS is frequently used as an indicator of efficiency in the ED, relying on this metric alone has notable limitations. The LOS is heavily influenced by external variables such as boarding times and availability of inpatient beds.^17^ These factors outside the control of ED processes can confound interpretations of LOS and obscure the true contributions of medical students. Furthermore, small sample size restricts the generalizability of findings and narrows the scope of potential outcome measures, limiting a comprehensive understanding of the role medical students play in ED efficiency and patient care.^8^ Additional studies have focused on evaluating relative value units (RVU) and patients seen per hour and have noted no significant differences at the expense of MS teaching.^18^,^19^ Although these metrics provide insight into productivity and throughput, they represent only a fraction of the complexity involved in assessing ED efficiency.

While the current literature mainly addresses the impact of resident physicians on ED throughput measures and patient care metrics, the limited studies pertaining to the impact of medical students have produced conflicting results. In this study we aimed to elucidate a more thorough understanding of the impact of a medical student through a more holistic lens of considering throughput, utilization, and outcomes metrics concurrently. This comprehensive evaluation could guide future resource allocation decisions, help enhance the balance between educational and operational priorities and ultimately improve both the quality of medical education and patient care in emergency settings.

## METHODS

### Data Source

We conducted a retrospective observational study at an urban, tertiary-care hospital in the United States to compare patient throughput, resource utilization, and clinical outcomes between encounters with and without MS involvement. This study was carried out in the adult ED of a large, community tertiary-care hospital, which accommodates approximately 95,000 patient visits annually. This adult ED evaluates patients >21, while those ≤21 years of age are evaluated in the pediatric ED and, therefore, were excluded from this study. The ED is staffed predominantly by board-certified emergency attendings, advanced practice practitioners (APP), and attendings who supervise medical students. All MS were in their fourth year on their mandatory core clerkship from the same medical school.

**Population Health Research Capsule**

What do we already know about this issue?

*Residents in the emergency department (ED) have variable effects on ED throughput, resource utilization, and clinical outcomes*.

What was the research question?

*What is the influence of medical students’ involvement on ED throughput, resource utilization, and clinical outcomes?*

What was the major finding of the study?

*We found overall doctor-to-disposition time (266.8 minutes vs. 242.9 minutes, P < .001) was longer with medical students, but for high-complexity patients, doctor-to-disposition times (275.2 vs 281.3 minutes, P =.02) with medical students were shorter*.

How does this improve population health?

*Calculating the “true” impact of a learner in the ED is difficult. More research is needed to balance learner interaction and bedside education with efficient, effective patient care*.

Patient encounters from January 1, 2022–December 31, 2023, were included in the study. We extracted data using a Crystal Report (SAP SE, Walldorf, Baden-Württemberg, Germany), a structured query language-based service that pulls data from the electronic health record (EHR) (Epic Systems Corporation, Verona, WI). Medical student involvement was determined through billing codes. As students were assigned within the ED to attending physicians for the duration of a shift, they were added to the care team function in the medical record for those attendings’ patients. This allows the billing/coders to be prompted to add the appropriate billing modifier (eg, GC modifier [treatment by a resident physician] on Medicare patients), which is a searchable, structured date-entry function in the EHR database. All clinical data were de-identified prior to analysis. All billing codes were derived from Current Procedural Terminology (CPT) codes and reflected the post-care evaluation of the level of service provided. Consistent with previous studies, low-complexity encounters contained CPT codes 99281, 99282; medium-complexity encounters contained CPT codes 99283, 99284; and high-complexity encounters were defined as CPT codes 99285, 99291.^20^,^21^ We applied the abstraction standards of Worster et al, which includes these elements: case-selection criteria definition; variable definition; abstraction forms (a database in which data was automatically abstracted from the EHR with no human review and all patient data scrubbed prior to entry into the database); blinding to hypothesis; medical record identified and scrubbed of identifying data in automated manner; a missing data plan, and institutional review board approval.^22^

### Study Design and Outcomes

Throughput metrics included arrival-to-disposition time (from ED arrival to entry of a discharge disposition in the EHR); door-to-triage time (from ED arrival to completion of assessment by a triage nurse); door-to-physician time (from ED arrival to an initial evaluation by a physician); and doctor-to-disposition time (from physician evaluation to entry of a discharge disposition in the EHR). We analyzed rates of discharge, hospital admission (including both inpatient and observation level of care), and transfer to another facility. Additional metrics included the percentage of patients who left against medical advice (AMA) and the rate of return to the ED within 72 hours.

We measured resource utilization by the number of diagnostic imaging studies per 100 visits, including computed tomography CT, MRI, plain radiographs, and ultrasound. Outcome metrics were evaluated through the admission rate, transfer rate, and Current Procedural Terminology (CPT) code complexity. We compared continuous variables using *t*-tests with bootstrap and categorical variables by chi-square tests. We reported continuous variables with mean and standard deviation, and the 95% confidence interval (CI) of mean difference. Data analysis was performed using Stata Statistical Software, Release 17.0 SE (StataCorp LLC, College Station, TX, 2021). This study was approved by the institutional review board of Rochester Regional Health.

## RESULTS

We analyzed patient encounters both with and without MS coverage across various complexity levels, as defined by CPT codes, from January 1, 2022–December 31, 2023. A total of 123,503 encounters were included in the analysis after excluding 42,578 due to age <21 and 23,945 seen only by APPs. Of these, 9,635 (7.8%) were MS-covered, and 113,868 (92.2%) were not (see Figure).

### General Metrics Across All Patient Groups

Across all encounters, door-to-physician time showed no significant difference between encounters with MS (28.1 minutes ± 38.6) and without MS (28.4 minutes ± 38.0; *P* = .44). However, door-to-triage time was significantly shorter with MS involvement (14.3 minutes ± 15.0 vs 15.7 minutes ± 27.7; *P* < .001). Arrival-to-disposition time (292.6 minutes ± 193.7 vs 270.4 minutes ± 532.8; *P* < .001) and doctor-to-disposition time (266.8 minutes ± 186.1 vs. 242.9 minutes ± 376.4; *P* < .001) were both significantly longer with MS involvement. Utilization measures per 100 visits showed significant differences in computed tomography (CT) (45.2/100 visits with MS vs 41.0/100 without, *P*<.001) and plain radiographs, ie, all radiographs of all types (47.1/100 visits with MS vs 44.7/100 visits with, *P* <.001) but not in MRI, ultrasounds, or portable chest radiographs (as a subgroup of all plain radiographs). Patient outcomes included higher admission rates (23.1% vs 18.1%; *P* < .001) and higher rates of leaving against medical advice (AMA) (1.7% vs 1.1%; *P* < .01) for encounters with MS. However, the discharge rate was lower (56.6% vs 66.7%; *P* < .001), with no significant difference in the rate of patients returning to the ED within 72 hours (6.3% vs 6.4%; *P* = .55) (Table 1).

### Low-Complexity Encounters

Patients with medical students experienced longer door-to-physician times (32.3 vs 28.9 minutes, *P* = .12) and arrival-to-disposition times (186.5 vs 145.9 minutes, *P* < .001). Utilization measures showed no significant differences in CT, magnetic resonance imaging (MRI), or portable chest radiographs, but slightly higher ultrasound use (0.7 vs 0.3 per 100 visits, *P* = .37). Discharge rates were notably lower (28.5% vs.54.5%, *P* < .001), although the return to ED within 72 hours did not differ significantly (Table 2).

### Medium-Complexity Encounters

For medium complexity encounters, all throughput measures were longer with MS involvement, notably arrival-to-disposition time (290.0 minutes ± 253.4 vs 262.8 minutes ± 440.3; *P* < .001) and doctor-to-disposition time (260.1 minutes ± 252.3 vs 236.1 minutes ± 439.3; *P* < 0001). Use of CT was significantly higher (13.7 vs 10.6 per 100 visits, *P* < .001), as were AMA rates; but the discharge rate was significantly lower with MS involvement (87.4% vs 90.8%; *P* = .001) (Table 3).

### High-Complexity Encounters

For high-complexity encounters, medical student patients experienced shorter door-to-physician times (26.6 vs 28.2 minutes, *P* < .001) and door-to-triage times (13.6 vs 14.5 minutes, *P* = .03). Arrival-to-disposition (301.1 minutes with a MS vs 307.7 without, *P* =.02) and doctor-to-disposition times (275.2 minutes with MS vs 281.3 minutes, *P*=.02) were also shorter for the MS group. Utilization of CT and MRIs showed no significant differences, but there was a trend toward higher CT use, which did not reach statistical significance. Admission rates were equivalent (34.4% vs 33.6%, *P* =.22), and discharge rates were lower (45.4% vs. 48.4%, *P* < .001), with no significant differences in rates of leaving AMA or returning to the ED within 72 hours (Table 4).

## DISCUSSION

By the concurrent analysis of multiple opportunity costs of a learner in the venue of a busy ED, we sought to derive more holistic data for leaders managing the staffing and supervision models of teaching EDs. Medical students appear to create a time inefficiency in disposition time metrics (arrival-to-disposition, doctor-to-disposition), which does not exist in door-to-clinician or door-to-triage times. Ambulatory patients are generally triaged by an advanced practice practitioner prior to physician/student involvement and, therefore, the student should not affect those metrics. However, it should be noted that the disposition time inefficiency is correlated with complexity of patient care: it is highest for both metrics in low-complexity patients, less so in medium-complexity patients, and is, in fact, a time efficiency with superior time-to-disposition measures in high-complexity patients. In short, the more complicated the patient’s care, the more efficient the care. This contradicts findings in Delaney et al but is seen in one of the two medical centers in Corey et al.^8^,^20^ Of note is that these studies occurred pre-COVID-19 pandemic, and post-COVID-19 pandemic, respectively, and differences in workflow from the pandemic cannot be separately evaluated. However, it is reasonable to assume that the added time a student takes to perform a history or perform a procedure on the less acutely ill patients and then present those findings to an attending accounts for the disparities at lower levels of acuity. In contrast, an attending may provide direct supervision and use the student as an extra data collector or procedural assistant on the higher acuity patients, resulting in time efficiency. From a staffing model perspective, it is possible that placing students predominantly in areas of high acuity may be a facilitator of care, while simultaneously preserving both the richness of educational opportunity that complex patients provide and the ability to provide significant direct supervision for feedback and evaluation purposes.

In all-comers across utilization measures, patients cared for by a medical student received more CT and plain radiographs. This is most likely due to the patient selection process for medical students, as the physician will choose patients with more interesting and complex pathophysiology. The less “interesting” patients would be selected to be seen by attendings only since those patients may not provide as rich a learning experience. The increased number of CT and plain radiographs may be associated with a student’s inexperience or lack of confidence in their examination skills, although this hypothesis cannot be proven in the format of this study. More resource-intensive testing (eg, MRI and ultrasound) rates were identical. However, on subgroup analysis, the only utilization rate that was significantly higher for medical student-covered patients was CT in the medium-complexity patient group. Low-acuity and high-acuity situations more typically follow a routine treatment algorithm/evaluation bundle and, therefore, these patients fall into an area where the inexperienced physician may generate more clinical certainty for themselves by relying on imaging. Additionally, this difference may be explained by an attending’s influence and clinical acumen on a medical student’s treatment plan. Further, higher acuity patients are more likely to get multiple radiologic images due to the need to evaluate their more complex state.

This utilization data contradicts previous studies of resident utilization data compared to attendings,^20^,^21^ although it is similar to the limited subset of data available from a 1999 French medical student strike.^11^ This may be explained by attendings having more direct impact on patient care with MS coverage rather than the model of encouraging residents to work toward independent practice.

Across patient outcome measures, MS-covered patients had higher admission rates and patients leaving AMA, although again this disappeared in high complexity encounters. As differences were contained within only the low- and medium-complexity patients, the prolonged time to disposition seen with MS coverage in those patients may explain this finding. There is limited literature with which to compare the data, but the medium-complexity patient data are similar to the small sample size in Jadhav et al, while contradicting Jadhav et al, for the low-complexity patients.^21^ This, therefore, seems an area for further research.

## LIMITATIONS

While this study included data from two years in a high-volume ED, there are several limitations. This was a retrospective, single-center study, which limits the generalizability of the findings. Of the patients covered without a medical student, care was provided in a heterogeneous manner, with multiple treatment pathways involving advanced practice practitioners working alone or in tandem with an attending, as well as attending physician care alone. Differential practice patterns may have affected the results. In addition, given these differential pathways, it was not possible to directly compare RVU generation between the models. Given that medical students have a tendency to see the most-complex patients, it would be reasonable to assume that RVU generation would be higher on a per patient basis, but this would have been confounded by patient selection biases. Additionally, due to nursing and bed shortages in the geographic region, boarding rates in the ED dramatically increased over the course of the study, creating a boarding crisis and subsequently leading to extreme shifts in length of stay and patients seen per hour. As these changes did not have any relationship with the presence of a medical student and were of a nature external to the ED, these metrics were excluded. Finally, due to a large number of patient visits, small effect sizes may have been statistically significant but not clinically significant (eg, five minutes).

## CONCLUSION

As reported in prior studies, calculating the “true” impact of a learner in the ED remains difficult; therefore, it remains an open discussion how to maximize learner interaction and bedside education with efficient, effective patient care. A model in which medical students predominantly care for higher acuity patients appears to be globally “less costly” than one in which they manage low- and medium-acuity patients. Validation of these findings in multicenter studies is necessary.

## Figures and Tables

**Figure:**
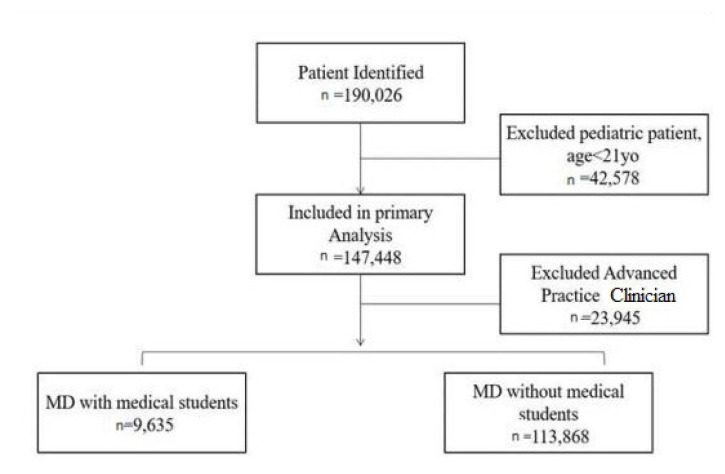
Patient selection flow chart. *MD*, medical doctor; *yo*, year old.

**Table 1:** Effect of medical students on emergency department throughout and utilization, comparative metrics across all patient groups with and without medical student coverage.

	Mean and SD in minutes with MS (n=9,635)	Mean and SD in minutes without MS (n=113,868)	*P*-value and 95% CI for mean difference
Throughput measures			
Door-to-physician time	28.1 (38.6)	28.4 (38.0)	*P* =.44 (95% CI: −1.1 to 0.5)
Door-to-triage time	14.3 (15.0)	15.7 (27.7)	*P* <.001 (95% CI −1.9 to 0.8)
Arrival-to-disposition time	292.6 (193.7)	270.4 (532.8)	*P* <.001 (95% CI 11.5 to 32.8)
Doctor-to-disposition time	266.8 (186.1)	242.9 (376.4)	*P* <.001 (95% CI 16.3 to 31.5)
Utilization measures			
CT/100	45.2 (76.5)	41.0 (73.7)	*P* <.001 (95% CI 2.7 to 5.7)
PR/100	47.1 (78.1)	44.7 (77.6)	*P* <.001 (95% CI 0.8 to 4.0)
MRI/100	1.4 (13.2)	1.3 (13.4)	*P* =.71 (95% CI: −0.2 to 0.3)
US/100	8.2 (28.5)	7.8 (28.2)	*P* =.25 (95% CI −0.2 to 0.9)
PCXR/100	9.5 (30.1)	9.4 (30.0)	*P* =.84 (95% CI −0.6 to 0.7)
Patient outcomes measures			
Admission rate (%)	23.1% (n=2,222)	18.1% (n=24,983)	*P* < .001 (95% CI 0.04 to 0.06)
Transfer rate (%)	0.3% (n=27)	0.3% (n=439)	*P* = .52 (95% CI: −0.001 to 0.0008)
AMA rate (%)	1.7% (n=162)	1.1% (n=1982)	*P* <.001 (95% CI 0.001 to 0.006)
Discharge rate (%)	56.6% (n=5,456)	66.7% (n=91,863)	*P* < .001 (95% CI −0.1 to −.09)
Return to ED within 72 hours rate (%)	6.3% (n=604)	6.4% (n=8,849)	*P* =.55 (95% CI −0.007 to 0.004)

*ED*, emergency department; *MS*, medical student; *CI*, confidence interval; *CT/100*, computed tomography per 100 visits; *PR/100*, plain radiographs per 100 visits; *MRI/100*, magnetic resonance imaging per 100 visits; *US/100*, ultrasound per 100 visits; *PCXR/100*, portable chest radiographs per 100 visits; *AMA*, against medical advice; *ED*, emergency department.

**Table 2:** Effect of medical students on emergency department throughout and utilization, comparative metrics across patient groups with and without medical student coverage for low-complexity encounters, hospital Current Procedural Terminology (CPT) codes.

	CPT 99281, 99282 (n=4,326)		

	Mean and SD in minutes with MS (n=305)	Mean and SD in minutes without MS (n=4,021)	*P*-value and 95% CI for mean difference
Throughput measures			
Door-to-physician time	32.3 (47.4)	28.9 (35.7)	.12 (95% CI −0.9 to 7.8)
Door-to-triage time	22.6 (20.8)	21.5 (21.7)	.41 (95% CI: −1.5 to 3.7)
Arrival-to-disposition time	186.5 (157.2)	145.9 (197.0)	<.001 (95% CI 17.9 to 63.2)
Doctor-to-disposition time	156.3 (153.1)	119.1 (196.2)	<.001 (95% CI 14.2 to 60.2)
Utilization measures			
CT/100	0.3 (5.7)	0.7 (11.8)	.59 (95% CI −1.7 to 1.0)
PR/100	0.3 (5.7)	1.1 (13.1)	.32 (95% CI −2.2 to 0.7)
MRI/100	0	0.02 (1.6)	.78 (95% CI −0.2 to 0.2)
US/100	0.7 (11.5)	0.3 (5.7)	.37 (95% CI −0.4 to 1.1)
PCXR/100	0	0.07 (0.03)	.63 (95% CI −0.4 to 0.2)
Patient outcomes measures			
Admission rate (%)	0	0.07% (n=3)	.63 (95% CI −0.004 to 0.002)
Transfer rate (%)	0.3% (n=1)	0	<.001 (95% CI 0.002 to 0.005)
AMA rate (%)	1.0% (n=3)	1.8% (n=74)	.28 (95% CI: −0.02 to 0.01)
Discharge rate (%)	28.5% (n=87)	54.5% (n=2,192)	<.001 (95% CI −0.32 to −0.20)
Returned to ED within 72 hours (%)	13.1% (n=40)	11.9% (n=478)	.52 (95% CI −0.03 to 0.05)

*MS*, medical student; *CI*, confidence interval; *CT/100*, computed tomography per 100 visits; *PR/100*, plain radiographs per 100 visits; *MRI/100*, magnetic resonance imaging per 100 visits; *US/100*, ultrasounds per 100 visits; *PCXR/100*, portable chest radiographs per 100 visits; *AMA*, against medical advice; *ED*, emergency department.

**Table 3:** Effect of medical students on emergency department throughout and utilization, comparative metrics across patient groups with and without medical student coverage for medium-complexity hospital Current Procedural Terminology (CPT) codes.

	CPT 99283, 99284 (n=39,121)		

	Mean and SD in minutes with MS (n=2,805)	Mean and SD in minutes without MS (n=36,316)	*P*-value and 95% CI for mean difference
Throughput measures			
Door-to-physician time	31.0 (41.8)	29.6 (38.8)	.08 (95% CI −0.2 to 2.9)
Door-to-triage time	15.2 (15.7)	15.6 (18.0)	.28 (95% CI −1.1 to −0.3)
Arrival-to-disposition time	290.0 (253.4)	262.8 (440.3)	<.001 (95% CI 8.7 to 41.7)
Doctor-to-disposition time	260.1 (252.3)	236.1 (439.3)	<.001 (95% CI 7.5 to 40.5)
Utilization measures			
CT/100	13.7 (47.7)	10.6 (42.3)	<.001 (95% CI 1.5 to − 4.7)
PR/100	32.8 (74.1)	32.2 (75.1)	.72 (95% CI −0.02 to 0.03)
MRI/100	0.07 (2.67)	0.1 (3.7)	.56 (95% CI −0.2 to 0.10)
US/100	0.6 (8.0)	0.4 (6.4)	.07 (95% CI −0.02 to 0.5)
PCXR/100	4.1 (19.9)	3.6 (19.2)	.25 (95% CI −0.3 to 1.2)
Patient outcomes measures			
Admission rate (%)	0	0.07% (n=24)	.17 (95% CI −0.002 to 0.0003)
Transfer rate (%)	0.1% (n=3)	0.2% (n=77)	.24 (95% CI −0.003 to 0.007)
AMA rate (%)	2.6% (n=73)	1.9% (n=691)	<.001 (95% CI 0.002 to 0.012)
Discharge rate (%)	87.4% (n=2,451)	90.8% (n=32,977)	<.001 (95% CI −0.05 to 0.02)
Returned to ED within 72 hrs (%)	8.4% (n=235)	7.8% (n=2,827)	.26 (95% CI −0.004 to 0.02)

*MS*, medical student; *CI*, confidence interval; *CT/100*, computed tomography per 100 visits; *PR/100*, plain radiographs per 100 visits; *MRI/100*, magnetic resonance imaging per 100 visits; *US/100*, ultrasound per 100 visits; *PCXR/100*, portable chest radiographs per 100 visits; *AMA*, against medical advice; *ED*, emergency department.

**Table 4:** Effect of medical students on emergency department throughout and utilization, comparative metrics across patient groups with and without medical student coverage for high-complexity hospital Current Procedural Terminology (CPT) codes.

	CPT 99285, 99291 (n=75,518)		

	Mean and SD in minutes with MS (n=6,407)	Mean and SD in minutes without MS (n=71,111)	*P* value and 95% CI for mean difference
Throughput measures			
Door-to-physician time	26.6 (36.4)	28.2 (38.0)	<.001 (95% CI −2.6 to 0.7)
Door-to-triage time	13.6 (14.2)	14.5 (33.7)	.03 (95% CI −1.7 to 0.7)
Arrival-to-disposition time	301.1 (160.0)	307.6 (218.4)	.02 (95% CI −12.0 to 1.0)
Doctor-to-disposition time	275.2 (147.4)	281.3 (212.9)	.02 (95% CI −11.5 to 0.8)
Utilization measures			
CT/100	61.8 (83.3)	59.8 (82.2)	.07 (95% CI: −0.2 to 4.1)
PR/100	56.3 (80.1)	54.8 (79.9)	.16 (95% CI −0.6 to 3.5)
MRI/100	2.1 (16.1)	2.1 (16.7)	.91 (95% CI −0.5 to 0.4)
US/100	10.4 (31.9)	10.6 (32.5)	.66 (95% C −1.0 to 0.6)
PCXR/100	14.0 (35.7)	14.8 (36.5)	.10 (95% CI −1.7 to 0.2)
Patient outcomes measures			
Admission rate (%)	34.4% (n=2,201)	33.6% (n=23,888)	.22 (95% CI −0.01 to 0.02)
Transfer rate (%)	0.3% (n=22)	0.5% (n=334)	.15 (95% CI −0.01 to 0.00)
AMA rate (%)	1.3% (n=85)	1.3% (n=955)	.91 (95% CI −0.003 to 0.003)
Discharge rate (%)	45.4% (n=2,910)	48.4% (n=34,447)	<.001 (95% CI −0.04 to −0.02)
Returned to ED within 72 hours (%)	4.9% (n=315)	5.4% (n=3,819)	.12 (95% CI −0.01 to 0.001)

*MS*, medical student; *CI*, confidence interval; *CT/100*, computed tomography per 100 visits; *PR/100*, plain radiographs per 100 visits; *MRI/100*, magnetic resonance imaging per 100 visits; *US/100*, ultrasound per 100 visits; *PCXR/100*, portable chest radiographs per 100 visits; *AMA*, against medical advice; *ED*, emergency department.
